# Intestinal ischemia following laparoscopic surgery: a case series

**DOI:** 10.1186/1752-1947-7-25

**Published:** 2013-01-21

**Authors:** Waleed Al-Khyatt, James D Thomas, David J Humes, Dileep N Lobo

**Affiliations:** 1 Division of Surgery, School of Graduate Entry Medicine and Health, University of Nottingham, Royal Derby Hospital, Uttoxetter Road, Derby DE22 3DT, UK; 2 Department of Radiology, Nottingham University Hospitals, Queen’s Medical Centre, Nottingham, UK; 3 Division of Gastrointestinal Surgery, Nottingham Digestive Diseases Centre NIHR Biomedical Research Unit, Nottingham University Hospitals, Queen’s Medical Centre, Nottingham, UK

## Abstract

**Introduction:**

Intestinal ischemia is a rare complication of laparoscopic surgery. Its prognosis depends on a high index of suspicion and effective early treatment.

**Case presentation:**

In the present report, we describe three cases where intestinal ischemia developed following laparoscopic surgery. Case 1 concerns a 52-year-old Caucasian man who developed large bowel ischemia following laparoscopic adjustable gastric band surgery. Case 2 concerns an 82-year-old Caucasian woman who developed fatal intestinal ischemia following laparoscopic cholecystectomy. Case 3 concerns a 58-year old Caucasian woman who developed right-sided lower intestinal ischemia following open cholecystectomy.

**Conclusions:**

Intestinal ischemia is a rare complication of laparoscopic surgery. The identification of high-risk patients is an essential primary preventive measure. A high index of suspicion is required to make an early diagnosis, which may help improve outcomes.

## Introduction

Intestinal ischemia is a rare complication of laparoscopic surgery. Its prognosis depends on a high index of suspicion and effective early treatment. However, the early signs and symptoms are entirely non-specific or even absent. Mesenteric ischemia has previously been reported after various laparoscopic procedures [1-14]. In this report, we describe two cases of large bowel ischemia following elective laparoscopic gastric banding (LAGB) and an elective laparoscopic cholecystectomy (LC), and a case of fatal small bowel ischemia following elective LC.

## Case presentation

### Case 1

A 52-year-old Caucasian man underwent LAGB for morbid obesity (body mass index (BMI) 49.7kg/m^2^). He had a medical history of diabetes mellitus, hypertension and obstructive sleep apnea. The procedure was performed according to the standard technique with maintenance of the intra-abdominal pressure at 15mmHg with a pneumoperitoneum time of 70 minutes. A calf muscle pump was applied during the procedure and our patient received low molecular weight heparin (LMWH) post-operatively. He had an uneventful post-operative recovery and he was discharged home within 24 hours. On the seventh post-operative day, he was readmitted with a two-day history of increasing abdominal pain and distension with multi-organ failure. He had leukocytosis (13 × 10^9 ^cells/L) and a raised lactate level of 5.5mmol/dL. An abdominal computed tomography (CT) scan showed intra-mural gas in the cecum and ascending colon, in keeping with ischemic bowel. On laparotomy, an ischemic right colon was identified with normal superior mesenteric artery (SMA) pulsation; therefore a right hemicolectomy was performed with an end ileostomy and removal of the gastric band. Post-operatively our patient required intensive care unit support for three weeks and renal replacement therapy. Post-operative histological examination demonstrated ischemic changes with no identifiable cause. He was discharged five weeks later.

### Case 2

**Figure 1:**
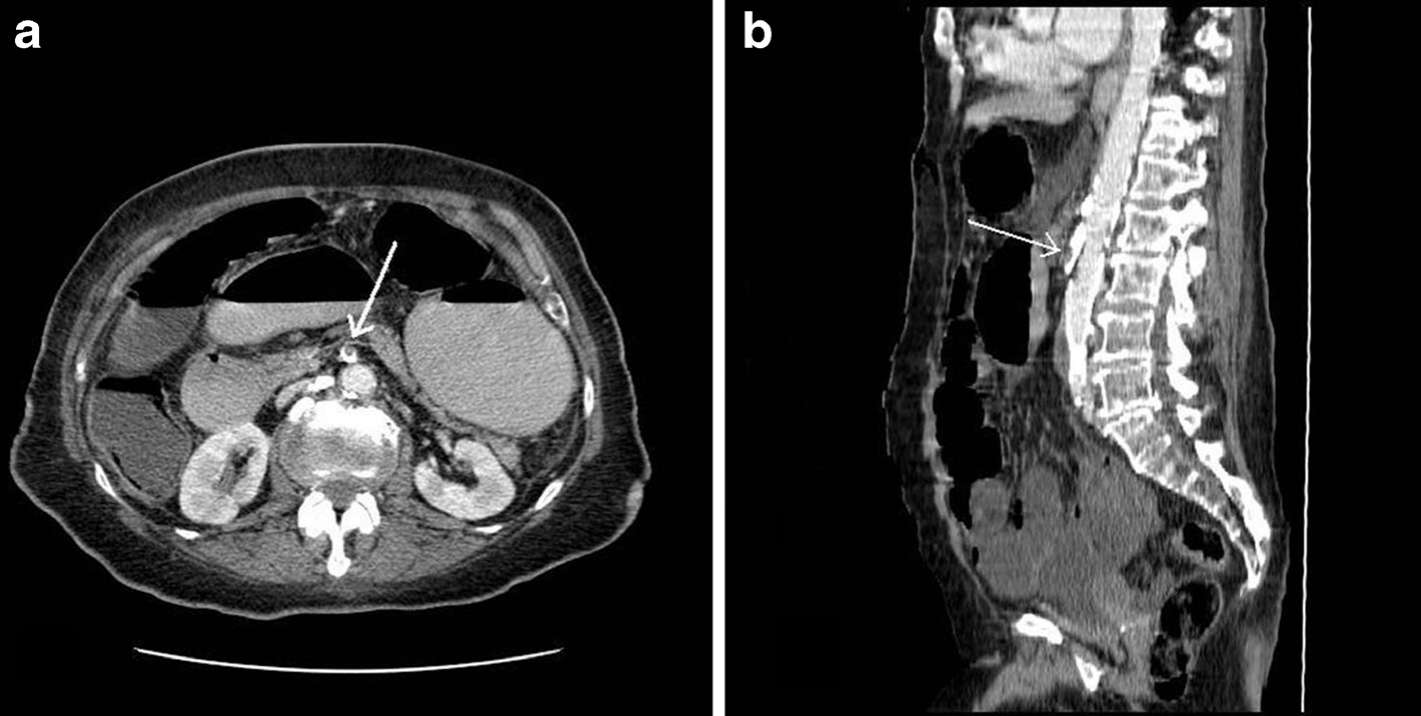
**(a) Axial and (b) parasagittal images from a computed tomography scan of the abdomen demonstrating a heavily calcified aorta and superior mesenteric artery.** No intravenous contrast can be seen within the superior mesenteric artery (arrow), suggesting total or subtotal occlusion.

An 82-year-old Caucasian woman underwent LC for symptomatic gallstones. Her medical history included hypertension and a previous transient ischemic attack on aspirin. LC was performed according to the standard technique with maintenance of intra-abdominal pressure at 12mmHg with a pneumoperitoneum time of 45 minutes. A calf muscle pump was applied during the procedure and our patient received LMWH post-operatively. Our patient was discharged on the first post-operative day. On the fifth post-operative day, she was readmitted with a history of severe generalized pain associated with vomiting and diarrhea. She had generalized abdominal distension and tenderness. Blood test results showed a raised C-reactive protein level of 300mg/L, and a raised lactate level of 6mmol/dL. An abdominal CT scan showed markedly dilated small and large bowel loops with a heavily calcified aorta and SMA. No intravenous contrast could be seen within the SMA (arrow, Figure 1) suggesting total or subtotal occlusion. On laparotomy, there was extensive ischemia of the stomach, small bowel and right colon. The mesenteric pulsation was undetectable. The operative findings were beyond surgical correction and she died six hours later.

### Case 3

A 58-year-old Caucasian woman with hypertension, diabetes and symptomatic gallstones was admitted for an elective LC. The procedure was performed according to the standard technique with maintenance of the intra-abdominal pressure at 12mmHg with a pneumoperitoneum time of 50 minutes prior to conversion to open cholecystectomy due to dense adhesions around the gallbladder. A calf muscle pump was applied during the procedure and our patient received LMWH post-operatively. On the third post-operative day she became quite unwell, and was hypotensive with a peritonitic abdomen and a raised lactate of 6mmol/dL. She was resuscitated and taken to theatre for a laparotomy, where an ischemic right-sided colon and distal terminal ileum was found. An extended right hemicolectomy was performed with an end ileostomy. She made a slow but uneventful recovery following her second procedure.

## Discussion

Acute intestinal ischemia is a rare complication following laparoscopic surgery. It has been described after LC (n=9) [1-3,5-9,13], inguinal hernia repair (n=1) [10], gynecological adhesiolysis and myolysis (n=1) [11], Nissen fundoplication (n=2) [4,12], and laparoscopic repair of incisional hernia (n=1) [14].

Normal intra-abdominal pressure (IAP) is 5 to 7mmHg and at an IAP of 12mmHg, renal functional impairment develops [15]. The profound effects of intra-abdominal hypertension (IAH) on intestinal circulation have been demonstrated in experimental studies [16,17]. The intra-abdominal perfusion pressure is the result of mean arterial blood pressure minus IAP [18]. Hence, a raised IAP due to pneumoperitoneum can predispose to splanchnic ischemia during laparoscopic surgery [19,20]. This initial ‘first hit’ causes diminished perfusion, mucosal acidosis and leads to the development of what is called acute intestinal distress syndrome [21,22]. In addition to this, IAH reduces cardiac output directly by compressing splanchnic venous return [23]. Moreover, the carbon dioxide (CO_2_) is absorbed into the circulation, resulting in hypercapnia, respiratory acidosis, and increasing the systemic vascular resistance secondary to the hemodynamic stress response (anti-diuretic hormone, renin activity, and catecholamines) [21,24-26]. Acute intestinal distress syndrome may be further triggered by either significant vascular narrowing or arterial thrombosis as in our second case [27]. Although these physiological changes create a theoretical risk of compromised intestinal blood flow, it is well tolerated by healthy adults with adequate cardiopulmonary reserve, with no clinical consequences.

Risk factors such as atherosclerosis and hepatic or renal impairment can predispose to ischemia [11]. Nearly 17 percent of patients undergoing LC have an American Society of Anesthesiologists (ASA) status of III or IV [28]. In the present report, our second patient had significant cardiovascular disease and CT scans showed extensive atherosclerosis of the SMA, which could have predisposed to this complication (Figure 1). However, an alternative explanation is that symptomatic episodes of mesenteric angina have been misinterpreted clinically as symptomatic gallstones [9]. However, our first and third patients had normal SMA pulsations on laparotomy and no causes of ischemia were found on post-operative histopathology in either case. Although acute intestinal ischemia is regarded as a rare complication of laparoscopic surgery, it is associated with 71 percent (12 out of 17 cases) mortality in reported cases, including this case series [1-14].

Diagnosis of acute intestinal ischemia requires a high index of suspicion. Patients commonly present with early non-specific symptoms of abdominal pain, nausea, vomiting with either diarrhea or delayed bowel action [29]. Prevention is best achieved by a thorough pre-operative assessment and early involvement of experienced surgeons in the selection of any anticipated difficult laparoscopic cases [28]. Gasless laparoscopy using abdominal wall lifting devices has been considered in an attempt to avoid the adverse effects of CO_2_ pneumoperitoneum that may occur in standard laparoscopy. This technique may be an alternative option in high-risk patients with cardiorespiratory diseases [30].

## Conclusions

Intestinal ischemia is a rare complication of laparoscopic surgery. It tends to occur mainly in high-risk patients with significant cardiopulmonary diseases or atherosclerosis. Proper pre-operative assessment to identify those patients at risk may help to prevent the development of this serious complication. A high index of suspicion is required to make an early diagnosis, which may help improve outcomes.

## Consent

Written informed consent was obtained from the patients (cases 1 and 3) or the patient’s next-of-kin (case 2) for publication of this manuscript and accompanying images. A copy of the written consent is available for review by the Editor-in-Chief of this journal.

## Competing interests

The authors declare that they have no competing interests.

## Authors’ contributions

WAK, DJH, and JDT prepared the manuscript. DNL outlined the manuscript’s layout and supervised the work. All authors read and approved the final manuscript.
